# Accurate Arrhythmia Classification with Multi-Branch, Multi-Head Attention Temporal Convolutional Networks

**DOI:** 10.3390/s24248124

**Published:** 2024-12-19

**Authors:** Suzhao Bi, Rongjian Lu, Qiang Xu, Peiwen Zhang

**Affiliations:** School of Mechanical and Electronic Engineering, Nanjing Forestry University, Nanjing 210037, China; bisuzhao@njfu.edu.cn (S.B.); lu_rjian@njfu.edu.cn (R.L.); 8230310326@njfu.edu.cn (P.Z.)

**Keywords:** electrocardiogram, arrhythmia classification, MB-MHA-TCN, data imbalance

## Abstract

Electrocardiogram (ECG) signals contain complex and diverse features, serving as a crucial basis for arrhythmia diagnosis. The subtle differences in characteristics among various types of arrhythmias, coupled with class imbalance issues in datasets, often hinder existing models from effectively capturing key information within these complex signals, leading to a bias towards normal classes. To address these challenges, this paper proposes a method for arrhythmia classification based on a multi-branch, multi-head attention temporal convolutional network (MB-MHA-TCN). The model integrates three convolutional branch layers with different kernel sizes and dilation rates to capture features across varying temporal scales. A multi-head self-attention mechanism dynamically allocates weights, integrating features and correlations from different branches to enhance the recognition capability for difficult-to-classify samples. Additionally, the temporal convolutional network employs multi-layer dilated convolutions to progressively expand the receptive field for extracting long-term dependencies. To tackle data imbalance, a novel data augmentation strategy is implemented, and focal loss is utilized to increase the weight of minority classes, while Bayesian optimization is employed to fine-tune the model’s hyperparameters. The results from five-fold cross-validation on the MIT-BIH Arrhythmia Database demonstrate that the proposed method achieves an overall accuracy of 98.75%, precision of 96.60%, sensitivity of 97.21%, and F1 score of 96.89% across five categories of ECG signals. Compared to other studies, this method exhibits superior performance in arrhythmia classification, significantly improving the recognition rate of minority classes.

## 1. Introduction

Arrhythmia is a disorder of heart rhythm caused by abnormal electrical activity, often manifesting as tachycardia, bradycardia, or atrial fibrillation (AF). These abnormal rhythms can impair the heart’s pumping efficiency, leading to inadequate blood circulation, and in severe cases, may result in myocardial ischemia, heart failure, or even sudden cardiac death [1]. With the global aging population, the incidence of arrhythmias is steadily increasing, posing a significant health threat to millions worldwide [2]. According to the World Health Organization (WHO), cardiovascular diseases are the leading cause of death globally, with arrhythmias playing a crucial role [3]. Particularly, severe types of arrhythmias, such as AF, ventricular tachycardia (VT), and ventricular fibrillation (VF), often progress without noticeable symptoms and can have serious clinical consequences when they occur.

**Figure 1:**
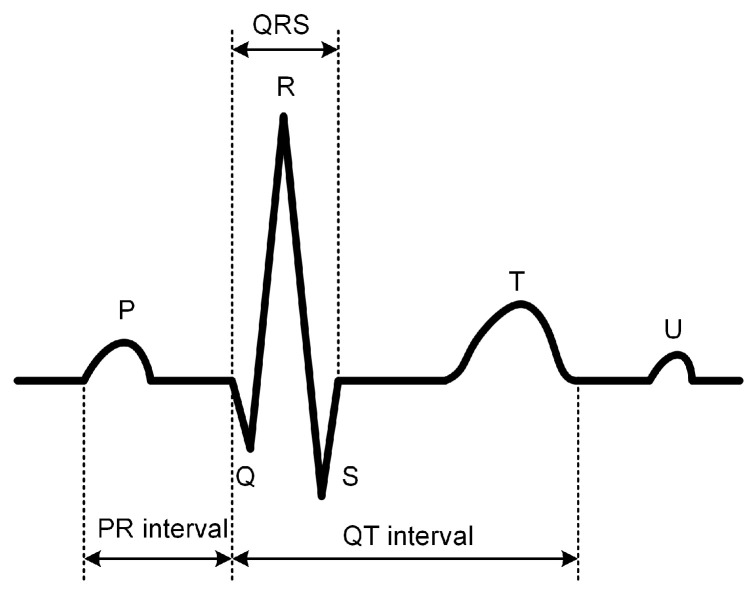
Standard ECG waveform.

Electrocardiography (ECG) is one of the most important tools for diagnosing arrhythmias, providing a graphical representation of the electrical activity of the heart with each heartbeat. Traditional arrhythmia diagnosis relies heavily on the analysis of ECG signals by experienced cardiologists, who identify subtle abnormalities within the ECG waveform to determine the type of arrhythmia. However, as a nonlinear and non-stationary biomedical signal, ECG is inherently complex and exhibits considerable similarity across different arrhythmia types, making manual analysis subject to significant inter-observer variability and diagnostic errors [4]. As illustrated in Figure 1, a typical heartbeat cycle shows distinct multi-scale features, with high-frequency components such as the QRS complex and low-frequency components such as the P and T waves, each corresponding to different phases of the heart’s electrical activity [5]. Furthermore, ECG signals are inherently weak, with amplitudes in the millivolt range and frequencies typically ranging from 0.05 Hz to 100 Hz. As a result, they are highly susceptible to noise interference, which can obscure the signal. Compounding the challenge, different arrhythmias may exhibit similar ECG patterns; for instance, the ECG morphologies of premature ventricular contractions (PVC) and fusion beats show significant overlap, further complicating accurate diagnosis.

In response to this issue, Ganan-Calvo and Fajardo [6] proposed a method to standardize heart rate variability (HRV) data by calculating the N-order forward moving average of RR intervals. They constructed N-dimensional normalized Poincaré plots and analyzed HRV by measuring the distance to the identity line. Additionally, they introduced primary variability (PV) and generalized angle (θ) metrics to quantitatively assess heart health [7]. This approach offers quantitative evaluation indicators that reduce subjectivity compared to manual ECG analysis, providing greater interpretability and more accurate assessment of cardiac conditions.

With the advancement of machine learning and deep learning in the medical field, classification algorithms for the automatic identification of arrhythmia types have become an essential tool to assist doctors in diagnosis. These algorithms enable faster and more accurate identification of arrhythmia types, reducing human error and improving diagnostic efficiency. Traditional machine learning-based methods for arrhythmia classification require manual extraction of representative features from raw signals, such as morphological features, statistical features, higher-order statistical features, and wavelet features. Feature selection techniques, such as Principal Component Analysis (PCA) [8], Recursive Feature Elimination (RFE) [9], and Chi-square tests [10], are then used to select the most representative features, reducing data dimensionality and avoiding overfitting. The selected features are subsequently fed into various classifiers for arrhythmia classification, including Artificial Neural Networks (ANN) [11], Support Vector Machines (SVM) [12], Random Forests (RF) [13], k-nearest neighbors (KNN) [14], Decision Trees [15], and Naive Bayes [16]. Due to their relatively simple and interpretable structures, traditional machine-learning methods have been widely used in early research on arrhythmia classification. However, their performance heavily depends on the quality of feature extraction, often resulting in suboptimal outcomes when features are extracted inadequately. Manual feature extraction also relies on domain experts’ prior knowledge, potentially leading to poor model generalization in the absence of sufficient expertise. Additionally, these methods exhibit limitations in handling nonlinear problems and imbalanced data, and they struggle to capture deep features in high-dimensional time-series data.

Deep learning methods utilize neural networks to automatically learn features from large datasets, reducing the complexity of manual feature extraction. These methods are well-suited for handling high-dimensional, nonlinear, and noise-rich time-series data. Compared to traditional methods, deep learning approaches offer strong generalization capabilities and are more effective in capturing complex patterns within ECG signals, making them powerful tools for feature learning. Hanbay [17] proposed a deep neural network (DNN)-based method for electrocardiogram (ECG) signal classification. This approach generates a hybrid feature vector by computing feature values and statistical characteristics and employs a denoising autoencoder (DAE) for unsupervised learning. The model is then fine-tuned using a softmax classifier and an active learning (AL) strategy. The results demonstrate that the proposed model achieves a 6.5% and 8.8% improvement in classification accuracy for VEB and SVEB, respectively, compared to traditional SVM methods.

Since ECG signals are one-dimensional time-series data, many studies [18,19,20] have employed one-dimensional convolutional neural networks (1D-CNNs), which slide convolutional kernels along the time axis to extract local features at each time step. This approach improves training efficiency and enhances the accuracy of arrhythmia classification. Acharya [21] first preprocesses the raw ECG signals by denoising, segmentation, and normalization. Synthetic data are generated using the Z-score method, followed by the implementation of a nine-layer deep convolutional neural network (CNN) for automatic classification of five types of heartbeats in ECG signals, achieving a classification accuracy of 94.03%. Kachuee [22] segments continuous ECG signals into 10 s windows, normalizes the amplitude, and segments the R-R intervals before feeding the signals into a convolutional neural network with 13 weight layers. The model achieves an average classification accuracy of 95.9% for arrhythmia detection. To further capture spatial or structural information in ECG signals, some studies have applied two-dimensional convolutional neural networks (2D-CNNs) to process 2D matrix data generated from multi-lead ECG signals or other 2D formats, such as time-frequency feature maps [23] and grayscale images [24].

Additionally, researchers [25,26] have introduced the self-attention mechanism, which captures global information by calculating the correlations between different positions in the sequence. This mechanism dynamically adjusts attention weights, enabling the model to focus on critical ECG features and improve classification accuracy. Multi-head attention [27] is an extension of self-attention that performs self-attention computations across multiple subspaces. The results from each attention head are then combined, allowing the model to simultaneously capture diverse information from different subspaces. This enables a more comprehensive understanding of the input signal by considering multiple aspects of it. Xu [28] proposed a multi-modal multi-attention network (MMNet), which first segments the ECG signals into individual beats, normalizes them, and converts them into images. Features are then extracted using a simplified ResNet-18 architecture. Finally, feature fusion is achieved through cross-attention and self-attention mechanisms. The model achieves an average accuracy of 97.72% on the MIT-BIH database.

Compared to convolutional neural networks (CNNs), recurrent neural networks (RNNs) and their variants, such as LSTM [29] and GRU, are more suitable for processing sequential data due to their recurrent structure, making them better at capturing long-term dependencies. Wang [30] introduced the Dual-Path Recurrent Neural Network (DPRNN), which segments single-lead ECG data into multiple parts. It iteratively models intra-segment and inter-segment sequences to extract comprehensive features. This method achieved 97.1% accuracy and an F1 score of 95.3% on the China Physiological Signal Challenge (CPSC) 2018 dataset. Mousavi et al. [31] proposed a heartbeat classification method combining a three-layer 1D CNN for feature extraction with a bidirectional LSTM encoder to capture both short- and long-term dependencies. The decoder generates heartbeat classifications, with bidirectional processing enabling the model to leverage both past and future contextual information. This approach achieved 92.57% accuracy and 88.94% sensitivity for supraventricular ectopic beats (S) and 99.50% accuracy and 99.94% sensitivity for ventricular ectopic beats (V) in the patient-based paradigm. Xu [32] proposed a model combining CNN and BiLSTM, where CNN is used to extract features from ECG signals, and the features are then input into a Bi-LSTM network for temporal modeling. The model achieves outstanding arrhythmia classification performance using pre-training and transfer learning strategies, with an F1 score of 95.92% and an accuracy of 95.90%. Essa [33] introduced a deep learning-based multi-model ensemble method for ECG arrhythmia classification. This approach combines CNN and LSTM models and employs a Bagging ensemble strategy along with fusion classifiers to effectively address the arrhythmia classification problem. By extracting both classical and deep features, and using the MIT-BIH database for training and testing, the method achieves an overall accuracy of 95.81%.

Leveraging the strong feature extraction capabilities and parallel computing advantages of CNNs, researchers [34] have proposed the Temporal Convolutional Network (TCN). By incorporating causal convolutions, dilated convolutions, and residual connections, TCNs offer a more flexible receptive field, stable gradient propagation, enhanced parallel computation, and the ability to capture long-term dependencies. Ingolfsson and his team [35] developed a lightweight TCN architecture that enables efficient deployment on wearable devices, achieving 94.2% accuracy on the ECG5000 dataset and improving the balanced accuracy score by 16.5%. Zhao [36] combined TCN with residual networks (ResNet), utilizing TCN’s strengths in time-domain analysis and ResNet’s capabilities in frequency feature extraction. This synergy resulted in an atrial fibrillation detection accuracy of 97% and an F1 score of 87%.

While deep learning methods have demonstrated great potential for arrhythmia classification, several challenges remain. Traditional CNNs are limited by fixed kernel sizes and receptive fields, making it difficult to capture features across varying time scales and long-range dependencies, thus constraining their ability to understand complex, diverse signals. Single-head self-attention mechanisms can only process inputs under a single attention distribution, making it challenging to capture features across different subspaces, and they often suffer from high computational complexity. Although the multi-head attention mechanism can capture global information from multiple perspectives, it tends to be less effective in processing local features and is more sensitive to noisy data. Additionally, RNNs and their variants are prone to gradient vanishing and exploding issues, and their sequential nature hinders parallelization, resulting in low computational efficiency. Dilated convolutions in deep TCNs can increase computational complexity and reduce robustness to noisy data, and they may be less effective at extracting short-term local features, thereby limiting their performance in arrhythmia classification.

Based on the limitations of existing research, this paper proposes a novel multi-branch, multi-head attention temporal convolutional network (MB-MHA-TCN) architecture aimed at improving arrhythmia classification accuracy and robustness with lower computational cost. Specifically, the multi-branch structure enhances the model’s ability to capture features across different time scales, while the multi-head attention mechanism dynamically allocates weights to improve the recognition of key ECG signal features. Additionally, the temporal convolutional network enables better capture of long-term dependencies. To address data imbalance, K-means clustering-based undersampling and SMOTE oversampling techniques are used, while Tomek Links optimizes the data distribution, further improving the accuracy of minority class recognition. During training, techniques such as focal loss, a custom learning rate scheduler, early stopping, and Bayesian optimization are applied to enhance model stability and generalization, ensuring optimal performance. The effectiveness of the model is validated through five-fold cross-validation and ablation experiments, demonstrating superior performance, particularly in the classification accuracy and robustness of the MIT-BIH Arrhythmia Database. Notably, the model shows significant improvement in minority class recognition.

The structure of this paper is as follows: Section 2 provides a detailed description of the proposed method and model architecture, Section 3 presents the experimental design and results analysis, and Section 4 concludes the study and suggests directions for future research.

## 2. Materials and Methods

**Figure 2:**
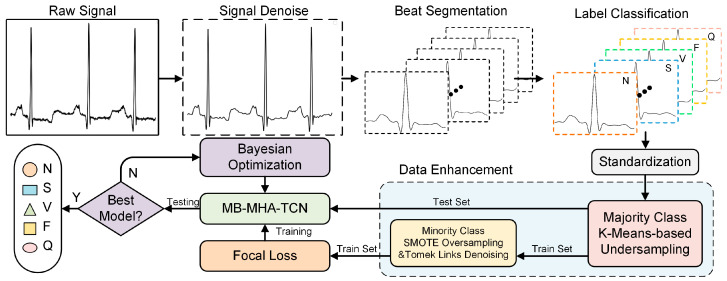
Flowchart of arrhythmia classification process.

Figure 2 illustrates the fundamental process of the proposed method. The main workflow begins with extracting ECG signals from the raw database, followed by signal filtering and noise reduction. Each heartbeat is then segmented and classified into five categories according to the AAMI standard based on the corresponding heartbeat labels. The data and labels are shuffled to form a dataset and subsequently standardized. The majority class is undersampled, and the dataset is split into training and test sets. For the minority classes in the training set, oversampling and data cleaning are applied. The processed data are then fed into our MB-MHA-TCN model. This model utilizes multi-class focal loss to enhance the weighting of minority classes, and Bayesian optimization is employed to fine-tune the model parameters, ultimately achieving optimal ECG signal classification.

### 2.1. MB-MHA-TCN Model

**Figure 3:**
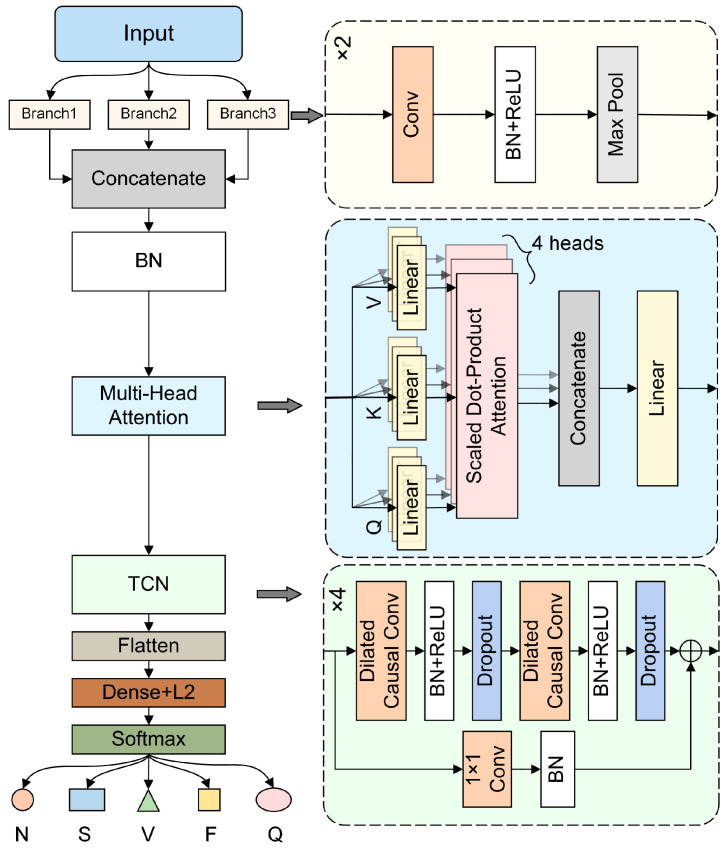
Architecture of the proposed MB-MHA-TCN model.

The proposed MB-MHA-TCN model integrates three key components in its architectural design: multi-branch convolution, multi-head self-attention mechanism, and temporal convolutional networks. The model architecture is illustrated in Figure 3, where the detailed internal structures of the three key components are expanded in corresponding color-coded boxes on the right. First, the multi-branch dilated convolution module extracts features across multiple temporal scales using different kernel sizes and dilation rates, providing a rich set of temporal information that lays the foundation for subsequent feature integration. Next, the multi-head self-attention mechanism dynamically adjusts the weights of each feature, capturing the relationships between different features and enhancing the model’s ability to identify key signal patterns. Finally, the TCN gradually expands the receptive field through multiple layers of dilated convolutions, effectively capturing long-term dependencies while maintaining low computational complexity through its lightweight design. The synergistic interaction among these modules enables the MB-MHA-TCN model to achieve superior feature extraction capability and robustness in arrhythmia classification.

#### 2.1.1. Multi-Branch Dilation Convolution

The multi-branch convolutional input module processes input data through three parallel convolutional branches, each utilizing different kernel sizes and dilation rates. This design enables the extraction of features across various temporal scales and feature dimensions, enhancing the model’s ability to capture complex patterns. The architecture is illustrated in Figure 3, with detailed parameters provided in Table 1. This structure is analogous to the human eye’s ability to perceive information at different resolutions, offering the model increased robustness and flexibility. This characteristic is particularly beneficial for handling ECG signals, as critical features corresponding to different cardiac events may manifest at different temporal scales.

In this model, the input consists of heartbeat data with a length of 250 samples, processed through three branches with convolutional kernel sizes of 4, 14, and 62, respectively, to capture multi-scale dependencies and features. The dilation rates are set to 1, 2, and 4, allowing for the expansion of the receptive field by inserting gaps between kernel elements without increasing computational complexity. This approach enables the convolutional layers to handle longer temporal spans without adding additional parameters.

Each branch contains two convolutional layers followed by pooling operations to ensure thorough feature extraction and compression. In the second convolutional layer, the kernel size is halved to further extract fine-grained features. By increasing the dilation rate, the network is also able to capture dependencies over longer temporal ranges. Compared to a deep single-branch network, the parallel, shallow structure of the multi-branch design allows for the extraction of rich features at relatively shallow levels, thereby reducing computational load and the number of parameters, which improves training efficiency.

Each convolutional layer is followed by a ReLU activation layer and a batch normalization layer to accelerate training and stabilize the model, mitigating issues like vanishing or exploding gradients. Max pooling layers are included for down-sampling, reducing computational complexity while retaining important features. Finally, a concatenate layer is used to merge features from all branches, integrating representations from different temporal scales. The concatenated features are then standardized to ensure stability during training.

#### 2.1.2. Multi-Head Self-Attention Mechanism

ECG data contains complex temporal dependencies. Therefore, after utilizing a multi-branch convolutional network, we employed an attention mechanism to further process and integrate the diverse features extracted from different branches, thereby enhancing the overall representation capability. This model incorporates the multi-head self-attention mechanism proposed by Vaswani et al. in their 2017 paper, “Attention is All You Need” [37]. Multi-head self-attention can be viewed as a combination of multiple self-attention heads, each obtaining independent queries (*Q*), keys (*K*), and values (*V*) through three linear transformations:

(1) $$Q_{i}=XW_{Q_{i}},K_{i}=XW_{K_{i}},V_{i}=XW_{V_{i}},i=1,2,\ldots ,h$$

here, $X\in R^{n\times d}$ is the input sequence, where *n* represents the sequence length, and *d* denotes the feature dimension. $W_{Q},W_{K},W_{V}\in R^{d\times d_{k}}$ are the trainable weight matrices, with $d_{k}$ being the internal dimension of the attention mechanism, and *h* indicates the number of attention heads.

The similarity between the queries and keys is computed using the dot product, followed by applying the softmax function to calculate the attention weights, yielding the output for each head:

(2) $${head}_{i}=softmax\left(\frac{Q_{i}K_{i}^{T}}{\sqrt{d_{k}}}\right)V_{i}$$

in this equation, $\frac{1}{\sqrt{d_{k}}}$ serves as a scaling factor to prevent excessively large dot product values that can lead to vanishing gradients.

The outputs from multiple self-attention heads are concatenated and then passed through a linear transformation to obtain the final output:

(3) $$MultiHead\left(Q_{i},K_{i},V_{i}\right)=Concat\left({head}_{1},{head}_{2},\ldots ,{head}_{h}\right)W_{O}$$

here, $W_{O}\in R^{hd_{k}\times d}$ is the output transformation matrix.

Compared to a single self-attention mechanism, multi-head attention enables the model to capture feature relationships from various perspectives and positions by independently calculating each head, allowing for a finer-grained capture of dependencies between different parts of the input sequence. Since each head operates on a lower feature dimension, the multi-head mechanism is more stable and easier to optimize during training compared to single-head attention. Additionally, the parallelism of multi-head attention enhances the efficiency of the model when processing long sequential data, effectively avoiding the common computational bottlenecks associated with long sequences in RNNs.

#### 2.1.3. Temporal Convolutional Network

Temporal Convolutional Networks (TCNs) leverage a series of one-dimensional convolutional layers and dilated convolutions to effectively capture temporal dependencies in sequences over extended time spans. In comparison to traditional RNN architectures, such as LSTM and GRU, TCNs provide several advantages, including parallel computation capabilities, more flexible receptive fields, faster training speeds, and reduced memory consumption when managing long sequential data. By integrating TCNs after the multi-branch network, we can further enhance the processing of diverse features, thereby improving the temporal relevance and expressiveness of the features over longer time ranges.

TCNs utilize causal convolutions, which ensure that the output at the current time step depends only on the data from the current and preceding time steps, thus maintaining strict temporal constraints and preventing information leakage. Nevertheless, purely causal convolutions still encounter challenges typical of conventional convolutional neural networks, such as the necessity of increasing kernel size to manage longer time series and the requirement to stack multiple layers linearly to capture longer dependencies. To address this, dilated convolutions introduce gaps between the elements of the convolutional kernel, exponentially expanding the receptive field and allowing the model to capture long-range dependencies without increasing computational complexity. Assuming $y\left(x\right)$ represents the output sequence and $x_{t}$ denotes the input sequence, the formula for dilated convolution can be expressed as follows:

(4) $$y\left(x\right)=\sum_{i=0}^{k-1}f\left(i\right)\cdot x_{t-d\cdot i}$$

where $f\left(i\right)$ is the convolution kernel, *k* is the kernel size, and *d* is the dilation rate.

To mitigate the vanishing gradient problem commonly encountered in deep networks, TCNs draw design inspiration from deep convolutional architectures like residual networks (ResNet) by incorporating residual connections. A residual connection is applied between every two layers of dilated convolution, allowing input information to bypass certain layers, forming a “shortcut”. Assuming $y\left(x\right)$ represents the output sequence, $x_{t}$ denotes the input sequence, and $F\left(x_{t}\right)$ denotes the operation of two dilated convolutions, the formula for the residual connection can be expressed as follows:

(5) $$y\left(x\right)=Activation\left(F\left(x_{t}\right)+x_{t}\right)$$

This approach stabilizes gradients during backpropagation, maintains consistency in the information flow, and aids the network in learning identity mappings, ultimately enhancing the model’s performance and generalization capability while mitigating the issue of network degradation.

To further reduce computational complexity without compromising the ability to capture temporal features, we adopted a compact TCN design by reducing the network depth. This lightweight design preserves the model’s capacity for capturing time-dependent relationships while decreasing computational demands and hardware resource requirements. Additionally, it mitigates the risk of overfitting, ensuring stability when handling noisy data, thereby achieving improved generalization performance and robustness.

To further reduce overfitting, we introduced L2 regularization after the TCN output. The fundamental principle is to add a penalty term to the loss function to constrain the size of the model weights. Let *L* denote our original loss function, then the new loss function with L2 regularization can be expressed as follows:

(6) $$L_{new}=L+\lambda \sum_{i=1}^{n}\omega_{i}^{2}$$

where *λ* is the parameter controlling the strength of regularization, and $\omega_{i}$ represents the *i*-th weight of the model. By adjusting the value of *λ*, we impose a greater penalty on larger weights, thereby promoting the model to learn smaller weights and enhancing its generalization capability.

### 2.2. Dataset and Preprocessing

This study utilizes the MIT-BIH Arrhythmia Database, developed through a collaboration between Beth Israel Hospital in Boston and the Massachusetts Institute of Technology and supported by PhysioNet [38]. Since its initial release in 1980, the MIT-BIH Arrhythmia Database has become a benchmark dataset for ECG signal analysis, widely employed in arrhythmia detection and classification research. The database comprises 48 dual-channel ECG recordings from various patients, each approximately 30 min long, with a sampling rate of 360 Hz. The data are meticulously annotated by multiple cardiology experts, encompassing approximately 110,000 heartbeats, including normal rhythms, ventricular ectopic beats, atrial ectopic beats, and other types. By utilizing this database, the study enables an accurate evaluation of the performance of the proposed arrhythmia detection algorithm.

ECG signals are characterized by their nonlinearity, low frequency, and weak signal strength. However, in real-world scenarios and public databases, ECG signals are often contaminated by various types of noise, such as baseline drift, power line interference, electromyographic (EMG) noise, and motion artifacts. These noises can obscure critical features of ECG signals, and their frequency bands often overlap with those of the ECG itself. Therefore, effective noise reduction is a crucial preprocessing step for further analysis. In this study, a Butterworth bandpass filter was applied to the ECG signals, with a passband frequency range set to 1–40 Hz, which encompasses the primary energy spectrum of the ECG signals. A low-pass cutoff frequency of 1 Hz was employed to remove baseline drift and other low-frequency noise, while a high-pass cutoff frequency of 40 Hz was applied to eliminate EMG and other high-frequency noise.

To further analyze ECG signals and prepare them for classification using neural network models, this study adopted a heartbeat-based segmentation method instead of fixed-length segmentation. Heartbeat-based segmentation ensures that each segment contains a complete cardiac cycle (including P waves, QRS complexes, and T waves), enabling the neural network to capture all critical features of the ECG signals, thereby improving the model’s learning and classification performance. In contrast, fixed-length segmentation may result in truncated cardiac cycles, leading to incomplete feature representation and increased redundant data. Since mature QRS detection algorithms are available for heartbeat segmentation, a detailed discussion of these methods is omitted in this paper. Using annotated data, we extracted the R-peak positions and corresponding heartbeat labels. To exclude unstable signal regions at the beginning and end of each recording, we retained the ECG data from the tenth heartbeat to the sixth-to-last heartbeat for analysis.

By analyzing heartbeat samples within the R-R intervals, we observed that the R-peak tends to be located in the latter half of the heartbeat cycle, near the P wave, rather than at the center. Based on this observation, we extracted 100 sample points before and 150 sample points after each R-peak, resulting in a fixed-length segment of 250 sample points, which corresponds to approximately 0.694 s at a sampling rate of 360 Hz. This approach ensures that even at lower heart rates (e.g., 60 bpm), the extracted segment captures the main waveforms of a complete cardiac cycle, effectively reducing data redundancy and simplifying the dataset.

The characteristics of ECG signals can vary significantly due to factors such as the subject’s age, lifestyle, and heart rate, leading to differences in the range and scale of the signals. To address this, this study employed the StandardScaler function to normalize the data. For a given feature *X*, the standardized value $X^{\prime }$ was calculated using the following formula:

(7) $$X^{\prime }=\frac{X-\mu }{\sigma }$$

In this process, *μ* represents the mean of feature *X*, and *σ* is its standard deviation. The StandardScaler function standardizes the data by transforming each feature to have a mean of 0 and a standard deviation of 1, using the calculated *μ* and *σ*. This transformation effectively eliminates differences in scale among various data sources and individual variations, ensuring consistency in amplitude and phase across ECG signals.

To ensure the generalizability of our results and facilitate fair comparison with other studies, we adhered to the AAMI (Association for the Advancement of Medical Instrumentation) standard for classifying heartbeats into five categories: normal beat (N), supraventricular ectopic beat (S), ventricular ectopic beat (V), fusion beat (F), and unknown beat (Q) [39]. Detailed information regarding each category under this standard is provided in Table 2.

### 2.3. Data Augmentation

As shown in Table 2, the MIT-BIH Arrhythmia Database exhibits a significant class imbalance issue, which may lead the model to favor the majority class during training. This bias can result in high overall accuracy while performing poorly in the detection of minority classes. To address this challenge, random undersampling of the majority class presents a straightforward approach; however, it risks losing important sample characteristics. Conversely, relying solely on oversampling techniques can lead to overfitting, and extensive oversampling generates a substantial amount of data, significantly increasing the complexity and duration of model training. More critically, in cases of considerable sample variation, newly generated samples may deviate from the actual data distribution, exacerbating the noise and outliers present in the original samples, which can negatively impact classification performance.

To tackle this issue, we propose a strategy that combines K-means clustering undersampling for the majority class, SMOTE (Synthetic Minority Over-sampling Technique) oversampling for the minority class, and Tomek Links for noise reduction to balance the dataset. K-means clustering is an unsupervised learning algorithm that partitions data points into *K* clusters by minimizing the Euclidean distance between each data point and the cluster centroid, as described by the objective function in Equation (8):

(8) $$J=\sum_{k=1}^{K}\sum_{x\in C_{k}}{\left\|x-c_{k}\right\|}^{2}$$

in this context, let *x* denote a data point in cluster $C_{k}$, $c_{k}$ represent the centroid of cluster $C_{k}$, and $\left\|x-c_{k}\right\|$ indicate the Euclidean distance between the data point and the cluster centroid. This method ensures that data points within the same cluster exhibit high similarity while data points across different clusters show significant variance. As shown in Table 1, the sample size of the majority class N is approximately 113 times that of the minority class F, highlighting a significant disparity in sample proportions. The method begins by randomly initializing *K* cluster centroids. The K-means algorithm is then applied to the majority class *N* samples by calculating the Euclidean distance between each sample and the cluster centroids, assigning each sample to the nearest cluster, and recomputing the cluster centroids as the mean of the assigned samples. This iterative process continues until convergence, defined as the change in centroid positions falling below a predefined threshold or reaching the maximum number of iterations. After clustering, the sample distribution within each cluster is analyzed to calculate the proportion of samples in each cluster, denoted as $P_{k}$. Based on $P_{k}$ and the desired total number of majority-class samples $N_{target}$, a target sampling count $S_{k}$ is assigned to each cluster. Subsequently, random sampling is performed within each cluster to select $S_{k}$ samples, ensuring that the reduced dataset maintains diversity and representativeness. Finally, the downsampled majority class samples are combined with the minority class samples to create a more balanced dataset. This approach effectively reduces the size of the majority class while preserving sample representativeness, minimizing feature loss, and avoiding oversimplification.

Given that the sample sizes for classes V and Q are relatively balanced, and the model performs well in these classes, we focused on oversampling the minority classes S and F, which account for only 2.5% and 0.7% of the total dataset, respectively. To address this imbalance, we employed the SMOTE method, a widely used oversampling technique for handling imbalanced datasets. SMOTE identifies *k* nearest neighbors for a minority class sample $x_{i}$ and then performs a linear interpolation between the minority sample $x_{i}$ and a selected neighbor $x_{j}$ to artificially synthesize a new sample $x_{new}$, which is then added to the training set. This algorithm generates new minority class samples by interpolating existing samples, thereby avoiding direct duplication of data and reducing the risk of overfitting. The synthesis formula is given by

(9) $$x_{new}=x_{i}+\lambda \cdot \left({x_{j}-x}_{i}\right)$$

where $x_{i}$ is the minority class sample, $x_{j}$ is one of its *k* nearest neighbors, and *λ* is a value in the range of $\left[0,\;1\right]$ that controls the distance between $x_{new}$ and $x_{i}$; $x_{new}$ lies on the line segment between $x_{i}$ and $x_{j}$.

However, since the SMOTE algorithm performs interpolation based on sample space, it may amplify noise and anomalies present in the dataset. To mitigate this effect after data augmentation, we applied the Tomek Links algorithm to clean the training samples and optimize the decision boundary. Two samples from different classes are considered a “Tomek Link” if they are nearest neighbors and have a short distance between them. The Euclidean distance between samples is computed using the following formula:

(10) $$d\left(x_{i},x_{j}\right)=\sqrt{\sum_{k=1}^{n}{\left(x_{i}^{k}-x_{j}^{k}\right)}^{2}}$$

where $x_{i}$ and $x_{j}$ represent two samples; $x_{i}^{k}$ and $x_{j}^{k}$ are the values of samples $x_{i}$ and $x_{j}$ on the *k*-th feature; and *n* is the number of features for each sample (the sample dimension). Upon detecting a “Tomek Link”, the majority class sample is typically removed, or both samples may be discarded when necessary to reduce class overlap and eliminate noisy instances from the dataset. This process can be repeated multiple times until a sufficient number of samples have been removed.

To avoid data leakage that could result in overly optimistic performance on the test set, thus obscuring the model’s true performance on real data, we first divided the dataset into training and test sets at an 80:20 ratio before oversampling. Subsequently, the training set was further partitioned into training and validation sets at an 80:20 ratio, ensuring that samples in the training and test sets remained independent. Table 3 presents the number and proportion of samples in each category within the augmented training, test, and validation sets.

## 3. Results and Discussion

### 3.1. Experimental Setup

The experiments were conducted using an NVIDIA GeForce RTX 3080 Ti GPU on a 64-bit Windows 10 system, with model development implemented using the Keras 2.4.3 and TensorFlow 2.4.0 frameworks.

During model training, we employed a custom learning rate scheduling strategy that combines warmup and exponential decay to ensure training stability in the early stages and improve convergence speed while later reducing the learning rate to fine-tune model parameters. The parameter settings of the strategy are shown in Table 4.

In the initial training phase, a warmup strategy was used to gradually increase the learning rate from a lower initial value (*initial_lr*) to the target learning rate (*target_lr*). This approach prevents abrupt parameter changes that could destabilize the model early in training. By gradually increasing the learning rate, the model can steadily adapt to a higher learning rate, accelerating early convergence. The learning rate update rule during the warmup phase is as follows:

(11) $$warmup\_lr=initial\_lr+(target\_lr-initial\_lr)\times \frac{step}{warmup\_steps}$$

where *step* is the current training step, and *warmup_steps* represents the total steps in the warmup phase, calculated as follows:

(12) $$warmup\_steps=epochs\times \left(\frac{len\left(X_{train}\right)}{batch\_size}\right)$$

where *epochs* denotes the number of complete passes through the training dataset, $len(X_{train})$ is the total number of samples in the training set, and *batch_size* is the number of samples used per training iteration. Following the warmup phase, the learning rate enters an exponential decay stage. This phase gradually reduces the learning rate to enable finer parameter adjustments and to prevent oscillations or deviations near optimal points. Specifically, after every fixed number of steps, denoted as *decay_steps*, the learning rate decays by a factor of *decay_rate* until it reaches a minimum threshold *min_lr*. The update rule for the decay phase is defined as follows:

(13) $$decay\_lr=target\_lr\times {decay\_rate}^{\frac{step-warmup\_steps}{decay\_steps}}$$

To prevent overfitting, we implemented an early stopping mechanism. Training is halted if the validation loss does not significantly decrease over several consecutive epochs, and the model reverts to the weights corresponding to the lowest validation loss. Additionally, to address the class imbalance, we adopted the focal loss function, which assigns higher weights to hard-to-classify samples. This effectively mitigates the class imbalance problem and improves classification performance for minority classes. The focal loss function, a modification of cross-entropy loss, incorporates a class balance factor *α* and a modulation factor *γ*, and is defined as follows:

(14) $$Focal\_Loss\left(p_{t}\right)=-\alpha_{t}{\left(1-p_{t}\right)}^{\gamma }log\left(p_{t}\right)$$

where $p_{t}$ is the probability that a sample belongs to its true class.

**Figure 4:**
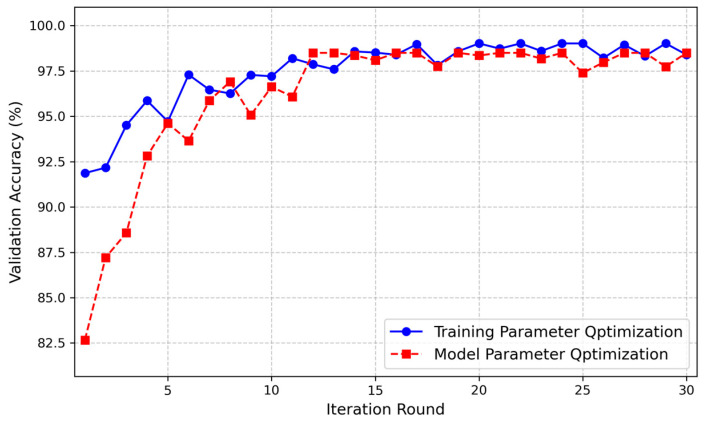
Validation accuracy under Bayesian optimization.

To achieve optimal hyperparameter combinations and improve model accuracy, we employed Bayesian optimization to fine-tune the critical hyperparameters of the model. Bayesian optimization constructs a surrogate model to approximate the objective function and uses it to determine the next evaluation point. In our optimization process, a Gaussian Process was used as the surrogate model, with the objective function set to the validation accuracy. The Expected Improvement (EI) acquisition function was applied to select the next set of hyperparameters for evaluation. The optimization procedure was configured with a maximum of 30 trials, with each trial executed twice. The final result of each trial was taken as the average of the two executions, aiming to mitigate the effects of randomness in data sampling and model initialization. Considering the model’s lightweight design and training efficiency, and based on existing experience and literature, we define the hyperparameter search space in Table 5. Due to the complexity of the model architecture and the large number of parameters involved in the training optimization process, we divided the hyperparameter tuning into two steps: first, optimizing the model parameters, followed by optimizing the training hyperparameters to achieve the best performance. The validation accuracy curve from the Bayesian hyperparameter tuning experiment is shown in Figure 4. It can be seen that this method converges rapidly and identifies the optimal hyperparameter combination (see Table 5), effectively enhancing the model’s performance.

### 3.2. Performance Matrices

This study assesses model performance using overall accuracy (*OA*), precision (*Pre*), sensitivity (*Sen*), and F1 score (*F1*), along with confusion matrices and AUC-ROC curves to visualize classification outcomes. These metrics are defined as follows:

(15) $$OA=\frac{TP+TN}{TP+TN+FP+FN}$$

(16) $$Pre=\frac{TP}{TP+FP}$$

(17) $$Sen=\frac{TP}{TP+FN}$$

(18) $$F1=2\times \frac{Pre\times Sen}{Pre+Sen}$$

where *TP* (true positive) indicates the number of correctly classified positive samples, *TN* (true negative) represents the number of correctly classified negative samples, *FP* (false positive) refers to the number of incorrectly classified positive samples, and *FN* (false negative) denotes the number of incorrectly classified negative samples.

### 3.3. Performance of the Proposed Method

For model validation and ablation studies, a 5-fold cross-validation method was employed. The dataset was randomly partitioned into five non-overlapping subsets, with each subset serving as a validation set in turn while the remaining four subsets were combined as the training set. This procedure was repeated over five iterations to obtain performance metrics, with the average values calculated to ensure stable and reliable evaluation results. Table 6 presents the macro-averaged performance of the model during 5-fold cross-validation. The results show average accuracy, precision, sensitivity, and F1 score values of 99.75%, 96.60%, 97.21%, and 96.89%, respectively, indicating the strong performance of the proposed method across multiple key metrics for arrhythmia classification. Moreover, the minimal variations between folds underscore the model’s stability and reliability. Additionally, the average AUC value is close to 1, suggesting a high discriminative capability and affirming the model’s generalization potential.

Due to the highly imbalanced nature of arrhythmia data, the F1 score, as a balanced metric that integrates precision and sensitivity, provides a more accurate reflection of the model’s effectiveness in handling imbalanced datasets. Therefore, when presenting the model’s training process and the performance across different classes, we selected the results from Fold 1—where the F1 score was the highest during 5-fold cross-validation—for visualization.

**Figure 5:**
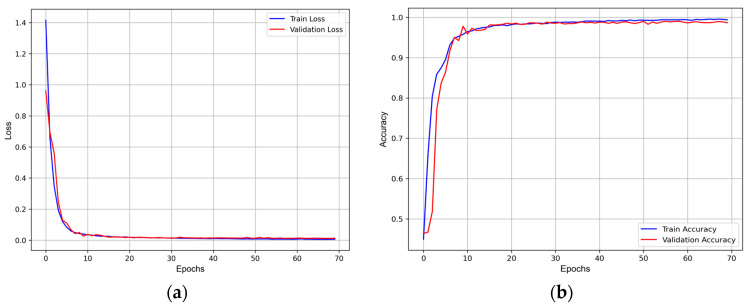
Loss and accuracy curves for Fold 1: (**a**) training and validation loss curves; (**b**) training and validation accuracy curves.

The loss and accuracy curves in Figure 5 both converge, with the training and validation curves nearly overlapping. This indicates consistent performance on both the training and validation sets, suggesting good generalization without signs of overfitting or underfitting. The smooth variation in loss and accuracy without significant fluctuations further confirms the stability of the training process and the appropriateness of the hyperparameter settings.

**Figure 6:**
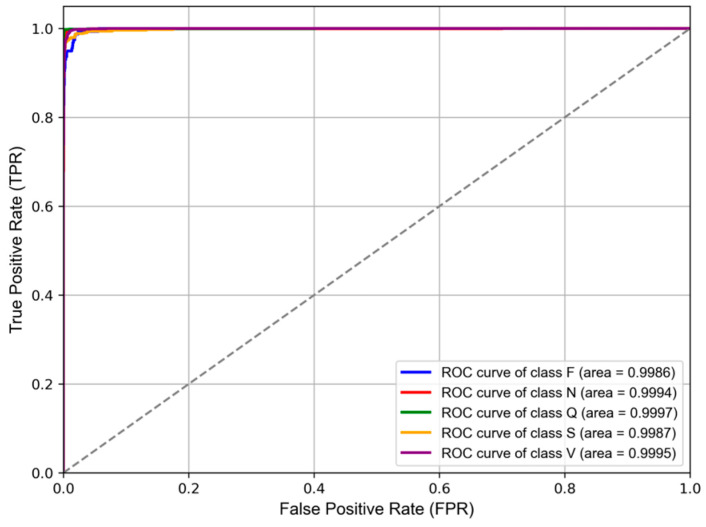
Receiver operating characteristic (ROC) curve for multi-class.

The ROC (receiver operating characteristic) curve is another critical tool for evaluating model classification performance. It plots the false positive rate (FPR) on the x-axis against the true positive rate (TPR) on the y-axis, illustrating the classifier’s performance across different thresholds. The AUC (Area Under the ROC Curve) value represents the area beneath the ROC curve, ranging from 0 to 1, with values closer to 1 indicating stronger discriminatory power. As shown in Figure 6, the ROC curves for all classes are concentrated near the top-left corner, and all AUC values exceed 99.8%. This indicates that the model is highly effective at distinguishing between different classes, demonstrating excellent generalization capability and robustness in handling complex datasets.

Table 7 presents the confusion matrix and classification metrics for each class in the test dataset of Fold 1. Overall, the model demonstrates excellent performance across most classes, achieving an overall accuracy (OA) of 99.02%, indicating strong generalization ability and stability. Notably, for class N (Normal) and class Q (Suspected), the precision, recall, and F1 scores are all close to 100%, with an extremely low misclassification rate. The F1 score for class V (ventricular) reaches 98.60%, although there is some minor confusion with class S (supraventricular) and class F (fusion). Among the 1391 V samples, the majority are correctly classified, with only six misclassified as S and five as F. While the overall performance for class S is slightly lower than that for class V, it remains robust, with an F1 score of 97.11%. Out of 532 S samples, only four were misclassified as class N and five as class V. Despite the limited sample size of class F in the dataset, its classification performance is slightly lower, yet the F1 score still reaches 93.97%.

### 3.4. Ablation Experiment

To analyze the contribution of each module within the proposed model and determine the specific impact of each component on performance, we selected TCN as the baseline model. Building upon this, we incrementally added key components from the proposed model, designing five sets of ablation experiments, as shown in Table 8. These experiments evaluated the detailed performance of the TCN, MB-TCN, MHA-TCN, and MB-MHA-TCN models.

The experimental results indicate that the baseline TCN model achieves an overall accuracy of 97.04% and an average F1 score of 93.77%, demonstrating its strong capability in arrhythmia classification. Due to its causal convolution structure, TCN can capture long-term temporal dependencies, leading to high sensitivity for majority classes such as N (98.31%) and Q (98.41%). However, TCN struggles with minority classes like S and F, particularly with a precision of only 76.97% for class F.

The introduction of a multi-branch structure in MB-TCN and MB-MHA-TCN allows the model to extract features across multiple scales, significantly improving overall performance, especially for minority classes S and F. Compared to TCN, MB-TCN shows a 1.08% increase in overall accuracy, a 1.69% increase in average F1 score, and improvements of 3.20% and 2.65% in the F1 scores of classes F and S, respectively. When compared to MHA-TCN, MB-MHA-TCN achieves a 0.48% increase in overall accuracy, a 0.95% increase in average F1 score, and improvements of 2.20% and 1.40% in the F1 scores of classes F and S, respectively.

The integration of the multi-head self-attention mechanism in MHA-TCN and MB-MHA-TCN enables parallel processing of multiple attention heads, focusing on signal features from different dimensions. This further enhances model performance. Compared to TCN, MHA-TCN achieves a 1.09% increase in overall accuracy, a 1.73% increase in average F1 score, and improvements of 3.22% and 2.83% in the F1 scores of classes F and S, respectively. Compared to MB-TCN, MB-MHA-TCN shows a 0.49% increase in overall accuracy, a 0.99% increase in average F1 score, and improvements of 2.22% and 1.58% in the F1 scores of classes F and S, respectively.

The ablation study results demonstrate that the incorporation of the multi-branch structure and multi-head self-attention mechanism in the proposed model significantly enhances feature extraction and classification capabilities for ECG signals, particularly improving the classification performance for minority classes.

Additionally, we compared the effects of focal loss (the proposed method) with Categorical Crossentropy Loss to explore the contribution of focal loss in addressing class imbalance. As shown in Table 9, we found that focal loss offered limited improvements in overall performance, with a 0.14% increase in overall accuracy and a 0.44% rise in the average F1 score. However, its primary advantage lies in handling minority class samples. With focal loss, the F1 scores for classes F and S improved by 1.27% and 0.76%, respectively. This improvement can be attributed to the smaller sample sizes of these classes, where traditional Categorical Crossentropy Loss tends to be dominated by majority class samples, leading to insufficient emphasis on minority class losses and making it challenging for the classifier to effectively capture their features. Focal loss addresses this issue by introducing two modulation factors that amplify the weight of hard-to-classify samples, encouraging the model to focus more on minority class samples during training, thereby significantly enhancing their classification performance.

### 3.5. Comparison of the Proposed Method to Other Previous Works

We compared the proposed model with several state-of-the-art methods that have shown excellent performance in arrhythmia classification in recent years (see Table 10) to validate the effectiveness of our approach. All methods in the table were trained and evaluated on the MIT-BIH Arrhythmia Database and adhered to the AAMI classification standard, ensuring the fairness and comparability of performance metrics. Additionally, these methods performed standard preprocessing steps, including normalization and heartbeat segmentation. Other preprocessing techniques, such as ECG denoising and data augmentation, are also summarized in the table.

As shown in Table 10, the proposed MB-MHA-TCN method outperforms existing mainstream ECG classification methods in several key metrics, including precision (Pre), sensitivity (Sen), specificity (Spe), F1 score (F1), and overall accuracy (OA). Compared to the CNN + BiLSTM model proposed by Mousavi et al. (2019), our method achieves improvements of 0.37%, 1.75%, and 1.17% in precision, sensitivity, and specificity, respectively, demonstrating significant advantages and robustness in handling minority class data and overall classification capability. However, the proposed method’s OA is slightly lower by 0.51%, which may be attributed to the bidirectional structure of BiLSTM. This architecture captures both forward and backward sequence information, enabling more comprehensive modeling of temporal dependencies, which provides an advantage in complex pattern recognition tasks. Nevertheless, the CNN + BiLSTM model has higher computational complexity and is more challenging to deploy on hardware. Its parameter size reaches 5.5 MB, which is 36 times larger than the proposed model’s size of 0.15 MB (float-32). Considering its lightweight design, the MB-MHA-TCN is more suitable for resource-constrained embedded systems and real-time application scenarios.

**Figure 7:**
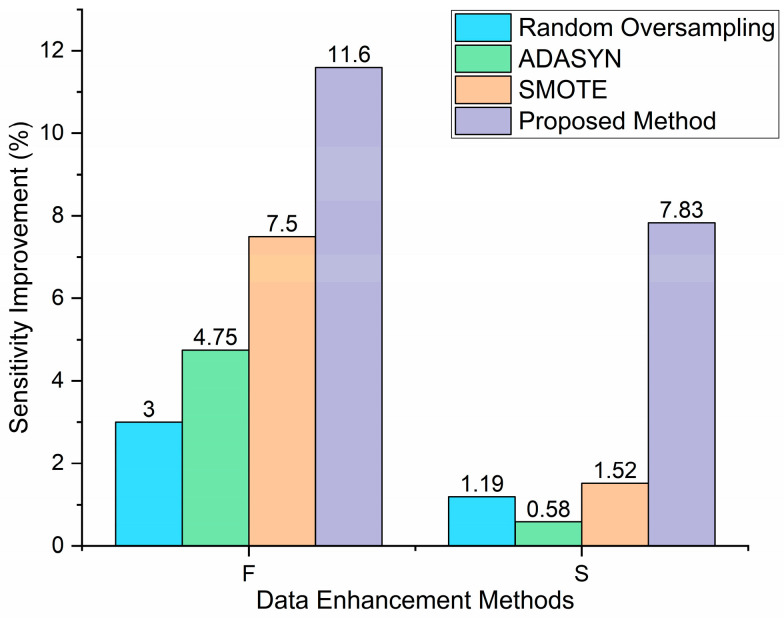
Sensitivity improvement of different data enhancement methods.

Figure 7 illustrates the impact of different data augmentation methods on arrhythmia classification performance for minority classes (F and S). The y-axis represents the sensitivity improvement percentage compared to the unbalanced dataset results. While traditional methods such as random oversampling, ADASYN, and SMOTE show some improvement in classifying minority samples, their gains are limited, especially in the sensitivity of class S. In contrast, the proposed data augmentation method demonstrates significant sensitivity improvements for both classes, with an increase of 11.6% for class F and 7.83% for class S. This indicates its superior effectiveness in addressing class imbalance and enhancing overall model performance.

## 4. Conclusions and Future Work

This paper proposes a method for arrhythmia classification based on a multi-branch, multi-head attention temporal convolutional network (MB-MHA-TCN) model aimed at enhancing the recognition of complex ECG signals and rare arrhythmia categories. The efficacy of each module is validated through ablation studies. To further address the class imbalance, this study combines K-means-based undersampling with SMOTE oversampling techniques optimized by Tomek Links to refine the data distribution. Additionally, focal loss is employed to amplify the model’s focus on minority classes. During training, various strategies are implemented, including a custom learning rate scheduler, early stopping, and Bayesian optimization, to enhance model stability and generalization capabilities, ultimately achieving optimal performance. Through five-fold cross-validation, the proposed method achieves an overall accuracy of up to 98.75% and an F1 score of 96.89% for the classification of five ECG signal categories according to the AAMI standard. This performance surpasses that of other studies, particularly in the significant improvement of minority class recognition rates. Future research will focus on integrating deep generative models, such as GANs or VAEs, to generate a more diverse minority class data. Additionally, transfer learning techniques will be explored to enhance the model’s generalization performance on diverse ECG datasets.

## Figures and Tables

**Table 1:** Parameters of the MB-MHA-TCN model.

Layer Type	Branch	Filter	Kernel/Pool Size	Dilation Rate	Stride	Activation Function	Batch Normalization	Other
Input Layer	-	-	-	-	-	-	-	250 × 1
Conv Layer 1	1	48	12	1	1	ReLU	Yes	-
Max Pooling 1	1	-	2	-	2	-	-	-
Conv Layer 2	1	64	6	1	1	ReLU	Yes	-
Max Pooling 2	1	-	2	-	2	-	-	-
Conv Layer 1	2	48	22	1	1	ReLU	Yes	-
Max Pooling 1	2	-	2	-	2	-	-	-
Conv Layer 2	2	64	11	2	1	ReLU	Yes	-
Max Pooling 2	2	-	2	-	2	-	-	-
Conv Layer 1	3	48	48	1	1	ReLU	Yes	-
Max Pooling 1	3	-	2	-	2	-	-	-
Conv Layer 2	3	64	24	4	1	ReLU	Yes	-
Max Pooling 2	3	-	2	-	2	-	-	-
Concatenate	-	-	-	-	-	-	Yes	-
MHA	-	-	-	-	-	-	-	4 heads
TCN Layer	-	6	8	-	-	ReLU	Yes	Dropout
Flatten Layer	-	-	-	-	-	-	-	-
Dense Layer	-	5	-	-	-	-	-	L2
Output Layer	-	-	-	-	-	Softmax	-	

**Table 2:** Detailed category information for MITDB using AAMI standard.

Category	Class	Number/% of Total ^1^
N	Normal beat (N)	73,520/68.5
	Left bundle branch block beat (L)	8030/7.5
	Right bundle branch block beat (R)	7187/6.7
	Atrial escape beat (e)	15/0.0
	Nodal (Junctional) beat (j)	216/0.2
SVEB	Atrial premature beat (A)	2454/2.3
	Aberrated atrial premature beat (a)	138/0.1
	Nodal (Junctional) premature beat (J)	69/0.1
	Supraventricular premature beat (S)	2/0.0
VEB	Premature ventricular contraction (V)	6854/6.4
	Ventricular escape beat (E)	106/0.1
F	Fusion of ventricular and normal beat (F)	785/0.7
Q	Paced beat (/)	6969/6.5
	Fusion of paced and normal beat (f)	977/0.9
	Unclassified beat (Q)	32/0.0
Total	-	107,354/100.0

^1^ The sample size in this column is the sample size after the preprocessing method proposed above rather than the sample size in the original dataset.

**Table 3:** Sample distribution in training, test, and validation sets after data enhancement.

Datasets	N	S	V	F	Q	Total
Training set	12,746	5561	4455	4817	5135	32,714
Test set	3074	532	1391	157	1572	6726
Validation set	2492	433	1108	128	1244	5405
Total/% of total	18,312/40.8	6526/14.6	6954/15.5	5102/11.4	7951/17.7	44,845/100.0

**Table 4:** Parameter settings for the learning rate scheduling strategy.

Strategy	Parameters	Value
Warmup	*initial_lr*	0.0001
	*target_lr*	0.0007
Exponential Decay	*decay_steps*	1500
	*decay_rate*	0.97
	*min_lr*	0.00001

**Table 5:** Search space and optimal hyperparameter configuration for Bayesian optimization.

Optimization Strategy		Hyperparameters	Search Space	Value
Optimum Model	CNN	*kernel_size_branch1*	[2, 16]	4
		*kernel_size_branch2*	[8, 32]	14
		*kernel_size_branch3*	[16, 128]	62
		*filt_*	[16, 64]	16
	MHA	*num_heads*	[4, 16]	4
	TCN	*kernel_size_tcn*	[4, 16]	8
		*layers*	[2, 5]	4
		*filt_tcn*	[6, 20]	10
Optimum Training Effect	Training parameter	*epochs*	[40, 200]	80
		*batch_size*	[32, 128]	64
		*drop_rate* ^1^	[0.1, 0.5]	0.4
	Focal Loss	*α*	[0.1, 2.0]	0.76943
		*γ*	[1, 5]	2

^1^ *drop_rate* denotes the proportion of input units that are randomly deactivated during training.

**Table 6:** Model classification performance based on 5-fold cross-validation.

Folds	OA *	Pre *	Sen *	F1 *	AUC *
Fold 0	98.68%	95.93%	97.21%	96.54%	99.68%
Fold 1	99.02%	97.58%	97.94%	97.76%	99.92%
Fold 2	98.59%	95.99%	97.06%	96.51%	99.83%
Fold 3	98.72%	96.56%	96.99%	96.77%	99.77%
Fold 4	98.75%	96.93%	96.86%	96.89%	99.80%
Average	98.75%	96.60%	97.21%	96.89%	99.80%

* These metrics represent the macro-averaged performance of the model across all classes without considering the proportion of each class in the dataset, thus preventing the majority class from dominating the overall performance.

**Table 7:** Classification performance based on Fold 1.

		Predicted Label					Performance (%)			
		N	S	V	F	Q	Pre	Sen	F1	OA
**True label**	**N**	3052	11	8	1	2	99.71%	99.28%	99.49%	99.02%
	**S**	4	520	5	1	2	96.47%	97.74%	97.11%	
	**V**	4	6	1373	5	3	98.49%	98.71%	98.60%	
	**F**	0	2	7	148	0	93.67%	94.27%	93.97%	
	**Q**	1	0	1	3	1567	99.56%	99.68%	99.62%	

**Table 8:** Ablation experiment results on classification performance of various categories.

Classes	Metrics ^1^	TCN	MB-TCN	MHA-TCN	Proposed Method ^2^
N	Pre	98.19%	99.26%	98.99%	**99.34%**
	Sen	98.31%	98.94%	99.12%	**99.40%**
	F1	98.25%	99.10%	99.05%	**99.37%**
S	Pre	89.75%	92.29%	93.93%	**96.71%**
	Sen	94.18%	96.96%	95.57%	**97.07%**
	F1	91.90%	94.55%	94.73%	**96.89%**
V	Pre	98.01%	98.37%	98.18%	**98.34%**
	Sen	94.36%	96.13%	96.42%	**97.96%**
	F1	96.15%	97.24%	97.29%	**98.15%**
F	Pre	76.97%	82.04%	84.01%	**88.92%**
	Sen	92.10%	92.74%	90.32%	**92.23%**
	F1	83.83%	87.03%	87.05%	**90.52%**
Q	Pre	99.02%	99.60%	99.39%	**99.68%**
	Sen	98.41%	99.20%	99.36%	**99.40%**
	F1	98.71%	99.40%	99.38%	**99.54%**
Average	Pre	92.39%	94.31%	94.90%	**96.60%**
	Sen	95.47%	96.79%	96.16%	**97.21%**
	F1	93.77%	95.46%	95.50%	**96.89%**
	OA	97.04%	98.12%	98.13%	**98.75%**

^1^ All assessment metrics in the table are averages obtained through 5-fold cross-validation. ^2^ The font in this column is bolded to highlight the classification performance of the proposed method categories.

**Table 9:** Cross-entropy and focal loss experiment results and comparison.

Loss Function	Classes	Pre	Sen	F1	OA
Categorical Crossentropy Loss	N	99.22%	99.43%	99.32%	98.61%
	S	96.28%	95.98%	96.13%	
	V	98.56%	97.43%	97.99%	
	F	87.18%	91.46%	89.25%	
	Q	99.48%	99.68%	99.58%	
Focal Loss	N	99.34%	99.40%	99.37%	98.75%
	S	96.71%	97.07%	96.89%	
	V	98.34%	97.96%	98.15%	
	F	88.92%	92.23%	90.52%	
	Q	99.68%	99.40%	99.54%	

**Table 10:** Comparison of classification performance between the proposed method and existing methods.

Author	Preprocessing	Approach *	Pre/%	Sen/%	Spe/%	F1/%	OA/%
Proposed method	Butterworth Bandpass Filter, K-Means, SMOTE, Tomek Links	MB-MHA-TCN	**97.58**	**97.94**	**99.75**	**97.76**	**99.02**
Wu et al., 2024 [40]	DPI, SMOTE	CNN + Transformer	-	88.1	-	82.6	95.7
Xu et al., 2023 [28]	Modal Conversion, Sample Enrichment	Multi-Head Attention	90.36	91.01	91.01	90.68	97.72
Essa et al., 2021 [33]	Baseline Correction, Low-Pass Filter	CNN + LSTM	74.97	69.20	94.56	71.06	95.81
Xu et al., 2020 [32]	Downsampling (125 Hz), Zero Padding	CNN + BiLSTM	96.34	95.9	-	**95.92**	95.9
Mousavi et al., 2019 [31]	SMOTE	CNN + BiLSTM	**97.21**	96.19	**98.58**	-	**99.53**
Hanbay et al., 2019 [17]	Median Filter, Low-Pass Filter	DNN	-	86.41	96.41	-	96.4
Kachuee et al., 2018 [22]	Zero Padding	1D-CNN	95.2	95.1	-	-	95.9
Acharya et al., 2017 [21]	Wavelet Filter (db6), Data Augmentation (Z-Score)	9-layer CNN model	-	**96.71**	91.54	-	94.03

* The empty cells under the performance metrics indicate that the corresponding data were not reported in the referenced studies. Bolded values are intended to highlight the best performance of the proposed model and the comparative methods, facilitating easier identification for readers. This is not intended to discredit the contributions of other studies.

## Data Availability

The data supporting the results of this study can be found in the MIT-BIH Arrhythmia Database, which is publicly available at PhysioNet (https://physionet.org/content/mitdb/1.0.0/) (accessed on 22 October 2024).
